# Hyalinizing Trabecular Tumor of the Thyroid Gland: A Case Report and Literature Review

**DOI:** 10.7759/cureus.37845

**Published:** 2023-04-19

**Authors:** Osama Alsogair, Abdullah A Alalawi, Ammar F Alzahim, Muhammad A Saleem, Faris M Aljohani, Lama S Alahmadi

**Affiliations:** 1 Surgery, King Salman Bin Abdulaziz Medical City, Madina, SAU; 2 Medicine, Al-Rayan Medical College, Madina, SAU; 3 Medicine, Taibah University, Madina, SAU

**Keywords:** paraganglioma like adenoma, histology, saudi arabia, thyroidectomy, differential diagnosis, thyroid tumor, fine needle aspiration biopsy, hyalinizing trabecular tumor

## Abstract

A hyalinizing trabecular tumor (HTT) of the thyroid gland is a very rare type of tumor. It is usually diagnosed incidentally during the examination for thyroid gland diseases that need thyroidectomy. Here we report a case of HTT in a 60-year-old male patient who presented with anterior neck swelling and underwent total thyroidectomy for a Bethesda category V nodule. The final histologic diagnosis of the left lobe was consistent with a hyalinized trabecular adenoma of the thyroid gland, or paraganglioma-like adenoma. We discuss the clinical picture and diagnostic approach, including the role of fine needle aspiration biopsy, and the pathologic features of HTT, with particular reference to the possible differential diagnosis.

## Introduction

Thyroid hyalinizing trabecular adenoma (HTA) was first described by Carney et al. in 1987 [1]. The World Health Organization calls it a borderline and rare follicular tumor with a trabecular pattern of cell growth and a lot of hyalinization inside the trabeculae [2]. This type of tumor makes up 1% of all thyroid tumors, is six times more likely to affect men than women, and is most common in people in their 50s [3,4]. This disease also has no symptoms, and if the lesion is smaller than 30 mm, it is usually found by accident during ultrasonography (US) [4]. On a histological level, tumor cells can be identified by yellow particles in the cytoplasm (staining that is positive for periodic acid-Schiff, or PAS) and a glass-like substance that is positive for collagen type IV [5]. Papillary thyroid carcinoma (PTC) can be differentiated from papillary thyroid cancer based on the fact that PTC tumor cells have Ki-67 on the cell membranes [6]. Even though this lesion usually has a benign course, it has been seen to spread to lymph nodes and other organs in a few patients [4,7].

## Case presentation

**Figure 1:**
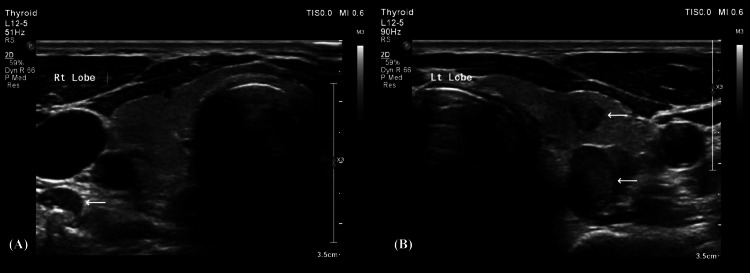
An ultrasound scan showing the right thyroid lobe (A) and the left thyroid lobe (B)

On October 12, 2021, a 60-year-old male patient presented with a mass on the right side of the neck. The patient had no compressive symptoms nor hypothyroid or hyperthyroid symptoms. He had no known chronic medical illnesses and had undergone a hemorrhoidectomy a few years ago. He was a nonsmoker with no family history of thyroid cancer. On examination, the thyroid gland revealed a palpable, firm and non-tender left thyroid nodule. Also, the palpable right thyroid nodule was firm and non-tender. There were no palpable cervical lymph nodes. An ultrasound examination revealed the right thyroid lobe measuring 1.8 x 1.6 x 3.8 cm and a small, rounded, calcified nodule near the isthmus that measured 0.7 × 0.8 cm (Figure 1). The left thyroid lobe was normal in size and measured 1.8 × 2 × 3 cm. It showed two echogenic nodules, the largest of which had solid and cystic components and measured 1.2 x 1.2 cm. The second one, the echogenic nodule, measured 0.4 x 0.5 cm. The laboratory work-up showed average complete blood count (CBC), renal profile, liver profile, and thyroid function tests.

**Figure 2:**
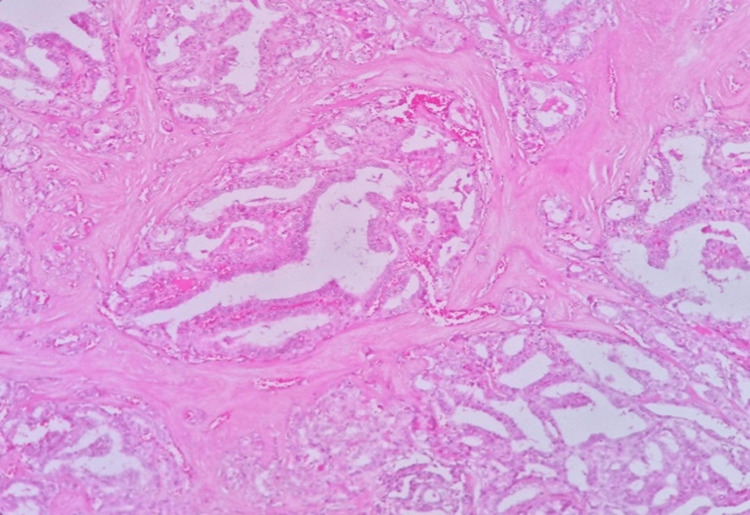
Histologic features of papillary structures lined by follicular epithelium displaying papillary carcinoma nuclear features at 40× magnification

Fine needle aspiration (FNA) showed a nodule suspected to be malignant, with a 60%-75% likelihood of malignancy. The patient agreed to have a total thyroidectomy after being counseled about his diagnosis and all treatment options. A microscopic examination of the right lobe (thyroidectomy) showed a focal lesion consisting of papillary structures and follicles lined by follicular epithelium displaying the nuclear features of papillary carcinoma (Figure 2).

**Figure 3:**
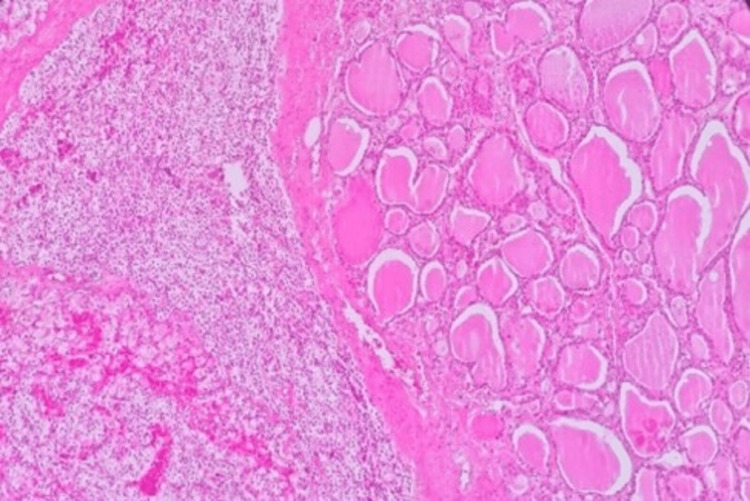
Histologic features of the left lobe showing a thin capsule separated by minimal fibrous stroma and intertrabecular hyaline and colloid at 10× magnification

The section was enclosed in a thin capsule with trabecular structures separated by minimal fibrous stroma. The intertrabecular hyaline and colloid were prominent, as shown in Figure 3.

**Figure 4:**
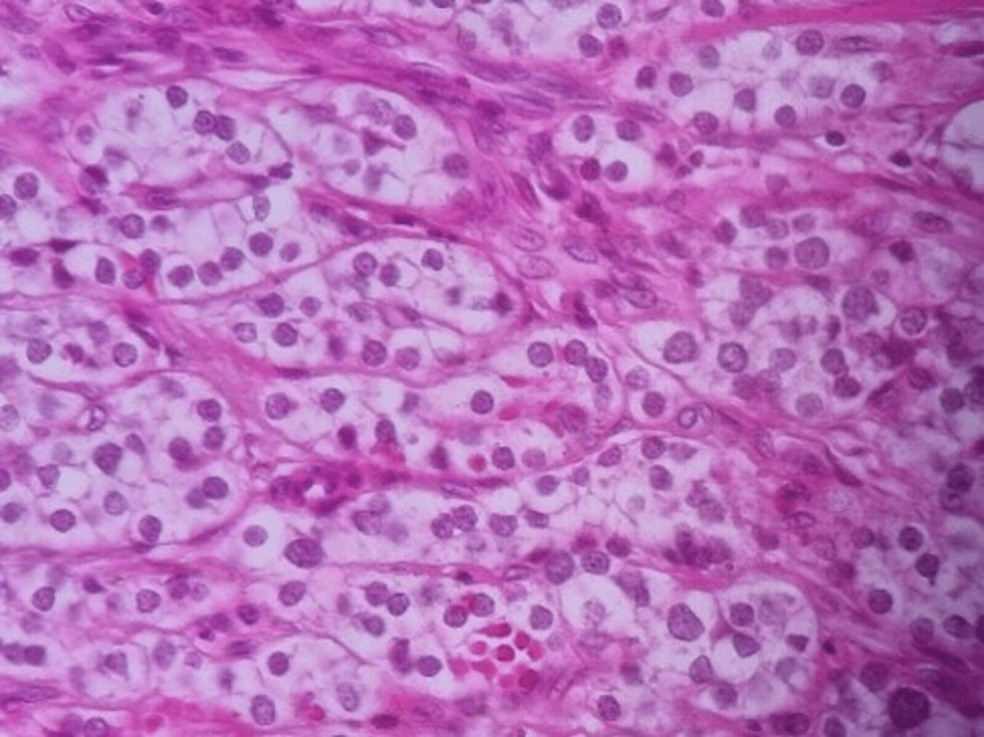
Histologic features of the left lobe showed polygonal to spindle-shaped cells at 40× magnification

Similar to paragangliomas, alveolar and Zellballen structures were present, partitioned by a sinus venous network. Tumor cells were polygonal, oval, or highly columnar, with an acidophilic or transparent cytoplasm. The nuclei were round or oval, with inconspicuous small nucleoli, noticeable grooves, and less common pseudoinclusions. The lesion's histology showed a trabecular and organoid architecture composed of spindle/elongated neoplastic cells with clumped nuclear chromatin and abundant eosinophilic cytoplasm. Extracellular hyalinization and significant stromal dystrophic calcification were also observed (Figure 4).

**Figure 5:**
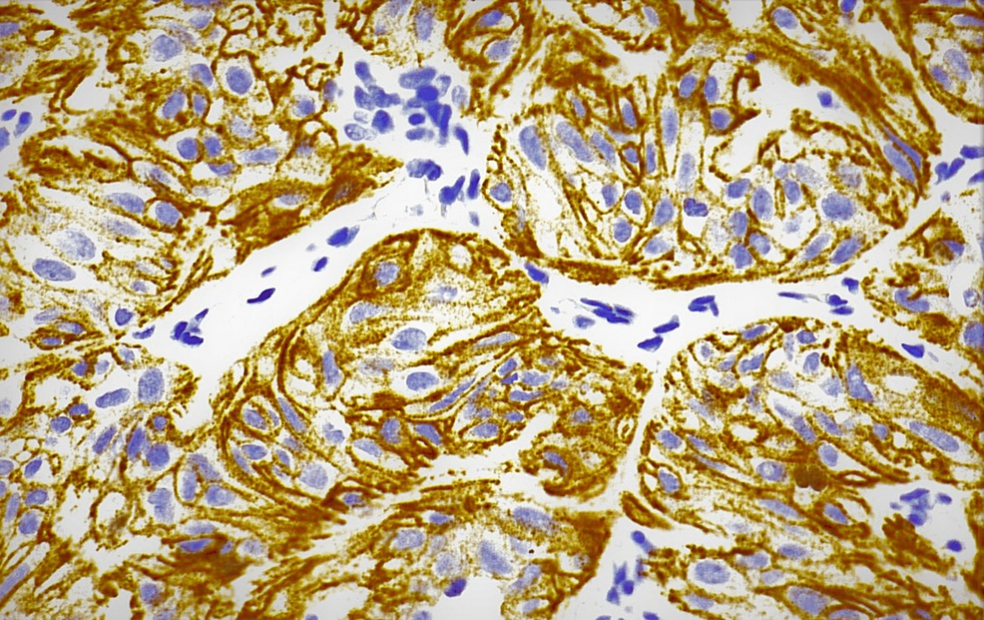
Thyroid membrane and peripheral cytoplasm reactivity (brown color) of Ki-67 immunohistochemistry staining

Immunohistochemistry was carried out. The tumor was thyroglobulin positive and chromogranin, calcitonin, and S-100 protein negative. M1B1 antibody (Ki-67) demonstrated strong cytoplasmic and membranous staining, which was especially helpful in identifying this case as the hyalinizing trabecular tumor (Figure 5).

In terms of the care plan, the patient started thyroxine tablets 125 mg once daily and was followed up on the 12th day post-total thyroidectomy. The goal of care was follow-up with ultrasound and thyroid function test. The patient came in for his follow-up and was doing well without any complaint. His thyroid stimulating hormone (TSH) was normal at 4.50 uU/Ml, and FT4 was 8.08 pmol/L. After three weeks of follow-up, the TSH level was within the normal range at 2.348 uU/mL and FT4 10.8 pmol/L. The patient came to the outpatient department free of any symptoms and was doing well with no new complaints. The ultrasound revealed no definite residual thyroid tissue in the thyroid beds, and only multiple enlarged lymph nodes in the right cervical and right submandibular regions, with a preserved hilum.

## Discussion

Since its initial description by Carney et al., the morphological diagnosis of HTT has remained challenging due to its overlapping histopathological characteristics and the ongoing controversy surrounding its underlying genetics, potential malignancy, and relationship with PTC [8]. In fact, Carney's initial description highlighted the fact that the trabecular pattern and nuclear characteristics (i.e., nuclear fissures and pseudoinclusions) overlapped those of PTC, posing diagnostic difficulties. Accordingly, in the WHO Classification of Tumours of Endocrine Organs, Fourth Edition, HTT was classified as a benign tumor [9,10].

Most people with HTT do not have any symptoms. The size and location of the tumor affect how a patient feels. As is the case with almost all thyroid nodules, the first step in figuring out what is wrong is to do an ultrasound scan and FNA. Absolute malignancy necessitates total thyroidectomy, whereas follicular neoplasms and suspicious nodules necessitate hemithyroidectomy to obtain specimens for the investigation of capsular invasion.

The histological hallmarks of HTT, which originate from follicular cells, include the presence of hyalinization and calcification of extracellular material, as well as the trabecular arrangement of neoplastic cells [11]. The nuclear-to-cytoplasmic ratio is often low in neoplastic cells, and nuclear grooves and pseudoinclusions are frequently observed. HTT is notoriously difficult to diagnose because of its similarities to more dangerous mimics such as PTCs and paragangliomas, all of which originate from follicular cells. Hypercellularity, cellular atypia with cytoplasmic invaginations, psammoma bodies, and nuclear pseudo-inclusions and/or grooves may all be present; these traits may overlap with one another [12]. Minimal cytologic atypia with low nuclear-to-cytoplasmic ratios, cellular aggregates around hyalinized material, and clumped or fine chromatin as opposed to optically transparent chromatin are all modest but distinguishing hallmarks of HTT [11].

It was hypothesized in 2012 that HTT could acquire mutations that would cause overexpression of RET/PTC and a subsequent malignant transition into PTC [12]. Complete resection, near-total thyroidectomy, or lobectomy are generally regarded as current therapeutic modalities, despite the lack of clarity on malignant potential. In a previous study by Carney et al., the largest known cohort of patients with hyalinizing trabecular tumors (n = 119) was studied over a span of 20 years [8]. Histologic nuclear and structural features showed psammoma bodies, pseudoinclusions, numerous grooves, trabecular structure, and organoid architecture features that were used as diagnostic criteria. Based on their findings, only one patient had capsular or vascular invasion and pulmonary metastases. Carney et al. found that most HTTs act like benign thyroid neoplasms; hence, they decided to call this type of tumor an "adenoma" [8].

Ki-67 (MIB-1) was identified by Hirokawa et al. as a promising marker for establishing a diagnosis of HTT [13,14]. In addition, they examined immunohistochemical staining patterns for cytokeratins (CK) (7, 14, 16, 17, 18, 19, 20) and high molecular weight (HMW) cytokeratins (1, 5, 10, 14) among 17 cases (12 unique cases of HTT and 5 unique cases of PTC) to further distinguish HTT from PTC. This was done to provide more evidence that HTT is not just a follicular form of PTC but rather a distinct entity in its own right. Their findings were noteworthy since the two entities showed nearly identical reactivity for CK 7 and 18; however, HTT showed varied reactivity for CK 19, while PTC showed robust expression.

## Conclusions

HTT is an uncommon and contentious thyroid tumor. It has a trabecular growth structure and a hyalinizing stroma. In addition to morphology, histochemistry and immunohistochemistry can be used to distinguish HTT from other thyroid cancers such as papillary thyroid carcinoma and medullary thyroid carcinoma. The prognosis of HTT is generally favorable, though a few instances may be followed by morphological malignant features or metastases.
